# A thematic analysis of caregiver engagement in adolescent substance use treatment

**DOI:** 10.1186/s13722-026-00669-z

**Published:** 2026-05-05

**Authors:** Trey V. Dellucci, Brielle L. Batch, Stephanie J. Strong, Amanda V. Broderick, Allyson L. Dir, Leslie A. Hulvershorn, Zachary W. Adams

**Affiliations:** 1 Department of Pediatrics, Indiana University School of Medicine, 410 W 10th St., Suite 1001, Indianapolis, IN 46202 USA; 2 Department of Psychiatry, Indiana University School of Medicine, 410 W 10th St., Indianapolis, IN 46202 USA; 3 Adolescent Behavioral Health Research Program, Indiana University School of Medicine, 410 W 10th St, Indianapolis, IN 46202 USA

**Keywords:** Adolescent, Substance use treatment, Caregiver, Engagement, Participation

## Abstract

**Background:**

While caregiver engagement is a critical component of adolescent substance use disorder (SUD) treatment, it is often limited in practice. Existing research on barriers and facilitators to caregiver engagement has predominately focused on clinician perspectives of caregiver engagement. This study sought to synthesize caregivers’ perspectives on their involvement in adolescent SUD treatment.

**Methods:**

Qualitative interviews were conducted with 15 caregivers (including parents and legal guardians) of adolescents who participated in an outpatient SUD treatment program in the past year. A hybrid inductive-deductive coding approach using an iterative and concurrent process was utilized to identify themes related to caregiver engagement.

**Findings:**

Thematic analysis identified three themes related to caregiver engagement: Caregiver Motivations for Treatment Initiation, Conceptualizations of Caregiver Involvement in Adolescent SUD Treatment, and Barriers and Facilitators of Caregiver Engagement.

**Conclusions:**

There is a need for early substance use screening and intervention development targeting caregivers’ motivation and psychoeducation on the benefits of caregiver engagement in adolescent SUD treatment and recovery.

**Supplementary Information:**

The online version contains supplementary material available at 10.1186/s13722-026-00669-z.

## Background

An estimated 2.2 million youth in the United States aged 12–17 met criteria for a substance use disorder (SUD) in 2023, yet only 35% of those received outpatient treatment for SUD [1]. Adolescents often lack insight into the severity of their substance use, partially due to ongoing social and brain development involved in decision-making, impulse control, emotion regulation, and reward processing [2]. Caregivers are a core component of most evidence-based interventions for adolescent SUDs by improving treatment adherence, reinforcing positive behaviors, and helping to identify early signs of relapse [3]. Despite their value in adolescent SUD treatment, caregiver engagement – broadly defined as the caregiver’s behavioral, attitudinal, and affective involvement in adolescent treatment – is often limited [4, 5].

The Health Belief Model and the Barriers to Treatment Model are two complementary theoretical frameworks that can be applied to the investigation of caregiver engagement in adolescent substance use treatment. The Health Belief Model focuses on individuals’ perceptions of health-related behaviors, emphasizing factors such as perceived susceptibility to problems, perceived severity of consequences, perceived benefits of treatment, and self-efficacy in completing treatment recommendations [6, 7]. In contrast, the Barriers to Treatment Model emphasizes internal and structural factors that impede or facilitate engagement, including logistical challenges, accessibility of services, family dynamics, and socioeconomic constraints [8]. Together, these frameworks provide a comprehensive lens for understanding motivations and obstacles that shape caregivers’ involvement in adolescent treatment, enabling researchers and clinicians to identify targets for intervention to enhance engagement and treatment outcomes.

Existing research investigating barriers to caregiver engagement in adolescent SUD treatment has often centered the perspectives of clinicians who specialize in SUD treatment (e.g., 9, 10, 11). Clinicians have identified caregivers’ unrealistic expectations about treatment, ambivalence or opposition to change, belief that the adolescent is solely responsible for their substance use, and caregiver guilt as barriers to caregiver engagement in adolescent SUD treatment [9–11]. Staff of adolescent SUD treatment programs have further described barriers to caregiver engagement including perceptions that adolescent substance use is episodic rather than chronic, beliefs that adolescent substance use is normal, and caregivers’ personal mental health and substance use concerns [12].

Research aiming to better understand caregiver engagement in adolescent SUD treatment from the caregiver perspective is notably sparse. These studies have primarily focused on caregiver motivations for and barriers to adolescent SUD treatment initiation. One qualitative study found that adolescent SUD treatment was most often initiated when change was desired by both adolescents and caregivers [13]; while others have described caregiver difficulty in finding adolescent SUD treatment options as a primary barrier to treatment initiation [11, 14]. Even less is known about caregiver engagement once adolescents are successfully connected to SUD care. One qualitative study identified the therapeutic relationship, provider experience and skill, and caregiver-inclusive treatment approaches as key indicators of adolescent SUD treatment quality [15]. Caregivers reported that attending treatment sessions improved their relationship with their adolescent, understanding of substance use, and reduced their stress.

More research focused on caregivers’ perspectives in adolescent SUD treatment is needed to better understand the factors that support or hinder caregiver engagement in adolescent SUD treatment. Insight into caregivers’ experiences could meaningfully inform improvements to existing evidence-based interventions and practices for adolescent SUD treatment and recovery. The current study addresses this gap through semi-structured interviews with caregivers of adolescents who recently completed SUD treatment, aiming to explore caregivers’ experience and engagement in treatment.

## Methods

### Setting and participants

Caregivers were recruited from an adolescent dual diagnosis clinic, which operates during traditional office hours and provides in-person and virtual outpatient treatment to youth who experience co-occurring substance use and behavioral health concerns. Adolescents typically present with an alcohol, cannabis, nicotine, or opioid use disorder – or multiple substance use disorders – and one or more co-occurring mental health conditions such as internalizing disorders (e.g., depression, anxiety), externalizing disorders (e.g., conduct disorder, oppositional defiant disorder, attention-deficit hyperactivity disorder), and/or post-traumatic stress disorder. Potential participants were identified based on a chart review of adolescents aged 13–18 who had completed treatment (i.e., met treatment goals, ended treatment with mutual agreement of family and clinical team) or discontinued therapy early (i.e., stopped attending sessions prior to completion of treatment goals or before recommended by clinical team) within an adolescent dual diagnoses clinic in the past year. Participants who discontinued treatment early were included to broaden the scope of caregiver experiences in treatment and to explore any potential differences in engagement between those who completed treatment and those who discontinued treatment. Caregivers were contacted by phone and those who expressed interest in the study provided verbal consent to participate and scheduled an interview with study staff.

The primary course of treatment in the adolescent dual diagnoses clinic from which participants were recruited involves an integrated set of evidence-based components including cognitive behavioral therapy (CBT), motivational interviewing (MI), contingency management (CM) and medication management when indicated (i.e., ENCOMPASS; [16, 17]). Psychotherapy modules are typically delivered across 12–16 weekly sessions, where most of the time is spent one-on-one with the adolescent and therapist. Caregivers are expected to participate in treatment by providing updates at the beginning and/or end of each session (updates, wrap-ups), and by participating in family sessions that are focused on substance use psychoeducation and communication skills training. Caregivers are also offered the opportunity to meet individually with a separate therapist for sessions focused on parenting related topics without their child present. The focus of the caregiver sessions is to provide the caregiver(s) with skills to manage their child’s substance use and mental health symptoms at home and to effectively cope with stressors related to their child’s substance use. Although these caregiver sessions are optional, caregivers are encouraged to participate in at least three caregiver sessions throughout the child’s course of treatment.

### Procedures

The Standards for Reporting Qualitative Research are followed in this report [18]. Interviews were conducted using a HIPAA compliant, web-based video conferencing software that allowed for audio recording. Interviews lasted no longer than 45 min, and participants were compensated with a $25 gift card. The study was determined to be exempt by the Indiana University Institutional Review Board because it involved minimal risk to the participants.

Participant sample size was determined using both the principles of information power [19] and thematic saturation [20]. Guided by the concept of information power, we considered the specificity of the study aim, the homogeneity of the sample, the use of established theoretical frameworks, and the quality of the interviews to assess whether the data collected were sufficiently rich and relevant to address the research question [19]. Concurrently, thematic saturation was evaluated through ongoing data analysis, with recruitment continuing until no new themes or meaningful insights emerged from the successive interviews [20]. Together, these complementary approaches ensured that the final sample size was both conceptually justified and empirically sufficient to support the study findings.

### Qualitative interview guide

The first author, a clinical psychologist with expertise in adolescent substance use treatment, initially developed the interview guide using the Health Belief Model [6, 7] and Barriers to Treatment Model [8] as guiding theoretical frameworks. Questions were structured to reflect key constructs such as recognition of child’s substance use, parenting challenges, self-efficacy, perceived obstacles and benefits for treatment, and motivation for change. Following the initial draft, the guide was refined through consultation with study authors, which included clinicians and researchers with expertise in adolescent substance use treatment. This group comprised a clinical research specialist, five clinical psychologists, and one psychiatrist who is board certified in both addiction medicine and child and adolescent psychiatry. Revisions were made to strengthen content validity, reduce redundancy, improve clarity and flow, and ensure alignment with the study’s conceptual framework and target population. The final interview guide is presented in Supplementary Material 1 and consists of questions assessing (a) recognition of child’s substance use, (b) the caregiver’s role in their child’s treatment, and (c) perceived barriers and benefits of being involved in their child’s treatment.

### Data analysis

Caregiver interviews were transcribed verbatim and analyzed using a hybrid inductive-deductive coding approach using an iterative and concurrent process [21–24]. An initial coding framework, guided by the Health Belief [6, 7] and Barriers to Treatment Models [8], was developed. Simultaneously, coders remained open to new themes that emerged throughout the iterative coding process. NVivo version 15 was used to organize and analyze data [25].

The analytic team included two licensed clinical psychologists with expertise in treating adolescent SUDs (TVD and SS) and a clinical research specialist (BB). Interviews were reviewed independently by members of the research team, who created memos of their initial impressions using open coding procedures [21, 26]. Coders then met to refine the initial code book and operational definitions. Each member of the analytic team coded two randomly selected transcripts. They then met to compare their coding, resolve discrepancies, and refine operational definitions, resulting in an updated codebook. Each analytic team member applied this codebook to a third transcript and met to discuss additional codes and refinement of operational definitions. The analytic team repeated this iterative process on a fourth and fifth randomly selected transcript, which yielded no additional themes or refinement of operational definitions and resulted in the final codebook.

All transcripts were assigned a primary and secondary coder to increase rigor through the process of triangulation [27]. Two coders independently coded each transcript using the final codebook. Discrepant codes were discussed within consensus meetings until 100% agreement was met. Quotes were grouped and reviewed by code, comparing content within and across transcripts, and organized into thematic categories [21–24].

## Results

### Participant characteristics

Interviews were conducted with 15 caregivers between August 2024 and March 2025. Participating caregivers included mothers (*n* = 11, 73.3%), fathers (*n* = 3, 20%), and one grandmother (6.7%). Most caregivers (*n* = 11, 73.3%) had a child who completed treatment. Given the small sample size, further participant characteristics are not reported to protect the confidentiality of the caregivers and their children.

### Emergent themes

Thematic analysis identified three themes describing caregiver engagement: Motivation for Treatment Initiation, Conceptualizations of Caregiver Involvement in Adolescent SUD Treatment, and Barriers and Facilitators of Caregiver Engagement. Each of these themes encompassed sub-themes which are discussed below.

#### Theme 1: Motivation for treatment initiation

Motivation for Treatment Initiation referred to the event that led the caregiver to seek out SUD treatment for their child. Three sub-themes regarding Caregivers’ Motivation for Treatment Initiation were identified including: Concerns for Adolescent Safety, Negative Academic Consequences, and the Caregiver’s Personal Experience with Substance Use. Results did not differ between families who completed treatment and families who discontinued treatment early.

*Concerns for adolescent safety.* Experiences of life-threatening events was a common theme for initiating treatment for substance use disorders among caregivers (*n* = 9, 60.0%). One caregiver described learning about their child’s substance use following a car accident:

She was in a car accident. It rolled over four times. There was six people in the car. They were all drunk… My daughter had to go to the hospital. Her knee was gashed open. She had to have like 17 stitches. (ID#41, discontinued treatment early)

Another caregiver described learning about their child’s substance use after having to save their child’s life through cardiopulmonary resuscitation (CPR):

He had a substance abuse problem for using opiates and I had to give him CPR for 15 minutes to keep him alive. That night he quit breathing…There was just the constant fear. So, you lose sleep. You can’t work. Like you you’re unable to do anything, because your main focus is to make sure that he’s breathing, and that he’s alive, that he’s not sneaking substances, and yeah things of that nature. (ID#52, completed treatment)

*Negative academic consequences.* Many caregivers reported looking for substance use treatment for their child after their child had experienced suspension or expulsion from school (*n* = 7, 46.7%). One caregiver shared:

My son, his freshman year of high school, was just having some trouble with substance abuse. He was expelled from school, making a lot of poor choices, just a lot of trouble at home, a lot of trouble at school, and a lot of trouble with friends. He was just kind of disengaged from things he used to like, so I did a lot of Google research trying to find any resources I could, which led me to you guys. (ID#56, completed treatment)

Some caregivers delayed treatment until their child acknowledged their substance use issues, often after repeated negative academic consequences (*n* = 4, 26.7%). One caregiver shared:

He was a freshman when that happened. He got caught vaping marijuana at school and initially, we kind of thought it was a one time, you know, ‘I messed up thing’. Then he ended up getting caught three times and the last time it was like two weeks of school left and they expelled him, and that day was kind of a turning point. That he realized and we did too that this had become a problem. This was not just him kind of experimenting or whatever he was doing it frequently and it was a problem. And that day he asked for help. (ID#61, completed treatment)

*Caregivers’ personal experience with substance use.* Two caregivers (13.3%) described initiating adolescent SUD treatment because they were familiar with the signs of problematic substance use from personal history. One caregiver shared:

She’s already showing behaviors, you know, that are concerning… I was terrified. Being a recovering addict myself, I really recognize that behavior. And I was scared, like my whole family, I don’t have much left, because they’ve all overdosed. (ID#37, completed treatment)

The second caregiver went on to describe that while they used substances in the past, opioids have only become more dangerous over time:

I’m only 16 years older than her, and that was like a different time. Even for me, you know. All we had was heroin back then. So, I’m like, I get, you know you want to experiment with things, but we live in a generation where experimenting will kill you.” (ID#13, discontinued treatment early)

#### Theme 2: Conceptualizations of caregiver involvement in adolescent SUD treatment

Caregivers Involvement in Adolescent SUD Treatment referred to caregivers perceived role in their child’s SUD treatment. Five sub-themes regarding caregivers’ involvement in adolescent SUD treatment were identified including: Facilitating Treatment Attendance, Attending Adolescent Sessions, Attending Caregiver Sessions, Monitoring and Means Restriction, and Reinforcing Adolescent Skills at Home.

*Facilitating treatment attendance.* Most caregivers, regardless of whether their child completed treatment, described facilitating treatment as a way to support their child in treatment (*n* = 12, 80.0%). For many this included driving their child to in-person appointments or ensuring they attended virtual sessions. For others it presented caregivers with a unique opportunity to learn more about their child’s experience in treatment from their child directly. As one caregiver described,

I think it was helpful for us to have that time together weekly, you know, going to the appointments, being there and having him be able to be open with me about things that were talked about during therapy. I think it was helpful to definitely just have that time together. (ID#56, completed treatment)

*Attending adolescent sessions*. Only caregivers of children who completed treatment described themselves as taking a more active role in their child’s individual appointments by participating in part of the child’s weekly therapy session (*n* = 8, 53.3%). Most commonly, caregivers would join the end of the therapy session to provide updates to the therapist or to learn about their child’s progress. One caregiver described perceiving the treatment program as a family therapy because they participated in every visit:

I feel like every session was a family treatment. Like [the therapist] would meet with [the child] for the first 45 minutes or so, and then the last 15 [minutes] would be together. So, I mean, I felt like I was a part of it the whole time. (ID#24, completed treatment)

*Attending individual caregiver sessions.* Most caregivers, regardless of whether their child completed treatment, attended at least one individual caregiver therapy session (*n* = 12, 80.0%). These sessions offered caregivers the opportunity to receive support and validation outside of the child’s individual session. One caregiver went on to say that while they originally thought caregiver sessions were unnecessary, these sessions helped them to readjust their perspectives on their child’s substance use:

[Child’s Therapist], encouraged those sessions, which was nice. You know, and I think there were times where I thought, ‘well, this isn’t really necessary. I don’t think that I need it. I know there’s parents dealing with so much worse.’ And, you know, I kind of went through it as this normal teenager behavior. I don’t know if I really need it. And then talking with [caregiver therapist] just made me feel so like, what you’re going through is not normal and it’s something you know that that would make anyone crazy. (ID#56, completed treatment)

Another highlighted that caregiver sessions encouraged communication and aligned expectations around substance use between both caregivers:

My husband and I were on two different pages. He was on the ‘oh it’s not a big deal that she went out and drank. I’m not going to ground her for it. You know, she was just trying to reach out and find friends.’ And I was like ‘I’m nailing her butt to the wall. I’m sick of this. She needs to straighten her butt up and so on and so forth.’ The counselor made my husband get on the same page with me. (ID#41, discontinued treatment early)

Although caregiver sessions were generally viewed as helpful, it was also common for caregivers to want more support that specifically focused on caregiver mental health and wellbeing rather than centering sessions on their child’s substance use. One caregiver noted that while they would have liked more support, they were unsure how feasible attending additional sessions would have been due to other obligations:

I think that parents are so consumed with helping their children that I don’t know if there’s something that you could give them to help them because they just spend all their time trying to make sure their kids turn out okay. But I don’t know what it would be, but I think that parents in general could probably use a little bit more support but at the same time that that takes time, and it takes coordinating and that’s really hard for parents, especially if they have other kids. If there was some way that parents could get more counseling or the ability to just talk through some things that might be helpful. I just don’t know how that would happen. (ID#50, completed treatment)

*Monitoring and means restriction.* Caregivers described their role in their child’s treatment and recovery as actively monitoring substance use, which included administering urine drug screens, maintaining regular communication about substance use, and restricting access to substances, (*n* = 14, 93.3%). This responsibility was reported by all caregivers of children who completed treatment, whereas it was relevant only for some of the caregivers of children who discontinued treatment early. One caregiver described the importance of setting expectations around substance use, particularly in settings where the child might be exposed to substances:

Her sister is turning 21 and we’re having a big party at our house on Saturday. So, we’ve talked about it. It’s like there’s going to be drinking here. Your sister’s 21 like this is, going to be fun. So, you know, you’re not allowed to drink whatever. So, we’re still talking about it a lot. (ID#24, completed treatment)

Others described limiting their child’s access to high-risk situations including both environments where substance use may be prevalent and limitations to time spent with individuals who engage in substance use:

I mean, we got rid of all inhalants in [the] house, all you know, potential or locked it up. We did that. We also stopped him from going over to his girlfriend’s house, where he could get access to that. We made the school aware. (ID#38, completed treatment)

One caregiver also described learning that random searches for substances was particularly important, because their child continued to have access to substances despite the caregiver’s efforts to restrict access in the home:

My husband and I wanted to believe that [child] was telling us the truth, and she wasn’t vaping, smoking, doing drugs and all this other stuff. And both counselors were telling us otherwise. They were telling us where to look in her room, where she was hiding the stuff and sure enough, we would wait for her to go to the bathroom or leave and there the stuff was. So, it was a major letdown that I felt like someone else knew my daughter better than me, but in the long run, it saved her life. (ID#41, ended treatment early)

*Reinforcing adolescent skills at home.* Most caregivers were aware that their child was expected to practice skills at home (*n* = 10, 66.7%). This awareness was more common among caregivers of children who completed substance use treatment and was reported by only one caregiver whose child did not complete treatment. Despite this, caregivers often allowed their child to complete homework assignments independently. One barrier to supporting skill practice at home was the caregiver’s effort to respect their child’s privacy and the integrity of the child-therapist relationship:

I would give him reminders [to complete homework]. I kind of left, you know, his sessions with his therapist between him and the therapist. I really wanted him to feel comfortable with her like she wasn’t sharing what they discussed. I really kind of left that between the two of them [child and child’s therapist]. (ID#56, completed treatment)

Another caregiver shared that they took an active role in their child’s substance use treatment by routinely checking in on how their child was doing emotionally and by checking in on cravings and urges to use substances:

During the off time, you know, between the sessions, just helping him like try to stay on track, you know, kind of check in with him basically. Like, how are you feeling? And, you know, are you, you know, I do actually say, do you miss doing it [using cannabis]? Like, you know, or do you feel like you’re past that point where you don’t miss it anymore? (ID#61, completed treatment)

Over time, these routine conversations evolved into daily walks that offered them the opportunity for protected quality time where both caregiver and child could share updates and lean on each other for support:

We started taking walks, which I think ended up being like super therapeutic for him. Hour-long walks and he loved it. It was fun. Like we would laugh, we would talk. It would be like, we would have really, really good conversation and, sometimes we would talk about this whole thing [child’s cannabis use], but then we would move on and just talk about all kinds of stuff, and we’d laugh and talk and sometimes, I mean, I think we got emotional talking about everything that had happened, but it was very therapeutic, I think, for both of us. (ID#61, completed treatment)

#### *Theme 3: Barriers and facilitators of caregiver engagement*

The third theme of caregiver engagement identified described Barriers and Facilitators to Caregiver Engagement, which included three sub-themes: Logistical Barriers and Facilitators, Treatment Components, and Individual Child Factors.

*Logistical Barriers and Facilitators.* Most caregivers, regardless of whether their child completed treatment, reported logistical barriers to engaging in treatment, including the clinic’s limited hours and the need to travel to the clinic (*n* = 11, 73.3%). These challenges were further exacerbated by caregivers’ work schedules and limited paid time off, leading many caregivers to miss appointments once their paid time off was exhausted. One caregiver shared:

I have a pretty flexible job, but it was still hard because they’re not like evening and weekend appointments. I was very limited in what I could work, which created a financial strain. (ID#57, completed treatment)

Another described the negative impacts of the additional travel time demands for attending appointments in person within the context of limited clinic hours:

I think some of it being that it’s located in [city], which essentially for a one-hour appointment makes it into about a two- and half-hour block of time. And to get him out of school. You know school goes till 4:00 pm and the last appointments are like at 3:00 pm, sometimes 4:00 pm. He’s always missing a chunk of time and that became difficult for him to keep up with his schoolwork because of that. (ID#53, completed treatment)

More than half of caregivers identified logistical factors that made attending appointments easier including scheduling medication visits on the same day as therapy sessions and having the option of virtual appointments (*n* = 8, 53.3%). These facilitators were more commonly indicated by caregivers of children who completed treatment than those whose children did not.

One thing for people to know ahead of time is the time commitment. I mean, it is once a week. We live about an hour and a half away. So, the therapist and the psychiatrist were so helpful at even doing virtual appointments, which was really nice for us. But yeah, it’s a huge commitment once a week to get drive [to city] and go. So that was probably the biggest challenge, you know, is just to make the weekly appointments for sure. (ID#56, completed treatment)

*Treatment Components.* Most caregivers identified aspects of treatment as facilitators of their involvement (*n* = 12, 80.%) and were more often identified by caregivers of children who completed substance use treatment compared to caregivers whose children did not complete treatment. These facilitators commonly included developing positive relationships with providers, improvements in caregiver-child relationships following family sessions, and receiving substance use psychoeducation. One caregiver described providers and staff as “super compassionate and super easy to talk to” (ID#20, discontinued treatment early). Several caregivers also described meaningful shifts in communication with their child because of the family sessions. For example, one caregiver shared:

Asking for [child’s] forgiveness for things we know that we’ve done wrong in turn [in response to her substance use]. So just like opening up the communication line and allowing her space to be really honest… We didn’t have conversations like that before (ID#24, completed treatment).

Caregivers emphasized that supportive interactions with providers fostered trust and comfort, while psychoeducation enhanced their understanding of substance use and equipped them to reinforce therapeutic skills and strategies at home.

*Individual Child Factors.* More than half of caregivers identified child-level factors that hindered treatment engagement, including the child’s willingness or motivation to participate and the child’s age (*n* = 8, 53.3%). These concerns were more frequently reported by caregivers of children who completed treatment, suggesting that even among treatment completers, navigating the balance between supporting child autonomy and managing low motivation remained an ongoing barrier to caregiver’s involvement. One caregiver illustrated this tension, saying:

We always felt that we were going to need some help, but [child] wasn’t willing up until that point… As he moves towards turning 18 or an adult, we have less control over this. We do know it needs to be his decision to stop this and we’re trying to point out consequences. You know, these things can happen [negative consequences of using substances] and just reiterating that. (ID#53, completed treatment)

## Discussion

This study adds to a small set of studies examining caregiver perceptions of their own engagement in adolescent SUD treatment [14, 15]. Thematic analysis revealed three themes related to caregiver engagement in their child’s SUD treatment including: Caregiver Motivations for Treatment Initiation, Conceptualizations of Caregiver Involvement, and Barriers and Facilitators to Caregiver Engagement. Motivation for Treatment Initiation described events that led to caregivers seeking SUD treatment for their child. Conceptualizations of Caregiver Involvement summarized caregivers’ perceived role in adolescent substance use treatment. Finally, Barriers and Facilitators of Caregiver Engagement summarized components of treatment that could be promoted or changed to improve accessibility for caregivers to actively engage in their child’s SUD treatment.

### Treatment initiation

There were no differences in motivations for treatment initiation among caregivers of children who completed treatment compared to those with children who did not complete treatment. Caregivers often initiated adolescent SUD treatment following a life-threatening event. For others, it was common for caregivers to delay treatment initiation until their child experienced both multiple negative academic consequences in the context of substance use and until their child communicated a desire to change their substance use with them. This finding replicates previous research, which found that most caregivers only initiated adolescent SUD treatment when negative consequences of substance use became apparent to both the caregiver and the adolescent [13]. This may indicate a gap in both adolescent and caregiver awareness regarding the broader impacts of substance use, extending beyond immediate and tangible outcomes such as medical emergencies. More subtle or long-term effects on mental health, social relationships, academic performance, and emotional development may be less apparent and more difficult to directly associate with substance use.

Enhancing caregivers’ knowledge about substance use and the particular risks for adolescents early may be crucial for identifying youth at risk for problematic substance use sooner. Mobile health (mHealth) education programs can serve as an effective tool to provide caregivers with practical information about substance use while also equipping them with the skills needed to engage in meaningful and supportive conversations with their children. For example, *Talk*,* They Hear You* is a publicly available mHealth application provided by Substance Abuse and Mental Health Services Administration [28]. The mHealth application offers substance use education, communication skills training, and screening tools for caregivers, with the goal of preventing or delaying the onset of adolescent substance use [28, 29]. Alternatively, universal substance use screening through Screening, Brief Intervention, and Referral to Treatment (SBIRT) in primary care and adolescent medicine clinics can also facilitate the early identification of adolescent substance use, provide timely education, and connect adolescents and their families with appropriate resources even before the onset of serious or severe substance use-related harms [30–33].

Even when caregivers recognize signs of adolescent substance use, a lack of adolescent motivation can pose a significant barrier to initiating treatment [13]. Brief interventions directed at caregivers may help to address this gap by increasing their knowledge of substance use and enhancing their ability to motivate adolescents to engage in substance use screening and treatment. Programs such as Community Reinforcement and Family Training (CRAFT) empower caregivers with practical skills to reduce their child’s substance use, increase their child’s motivation to initiate SUD treatment, and support their own emotional wellbeing [34–36]. By targeting caregivers first, these interventions can function as a valuable pre-intervention strategy to prepare and encourage adolescents to participate more fully in subsequent substance use assessments and treatment.

### Conceptualizations of caregiver involvement

Themes regarding caregivers’ involvement in their child’s treatment aligned broadly with behavioral domains of treatment engagement [5, 37]. However, caregivers differed in how they conceptualized their role within SUD treatment. Caregivers of children who did not complete treatment commonly described their role as primarily facilitating their child’s attendance. Meanwhile, caregivers of children who completed treatment further described their role as needing to attend weekly sessions alongside their child, monitoring substance use at home, and reinforcing therapeutic skills outside of treatment. Consistent with the Health Belief Model [6, 7], these differences may reflect varying levels of perceived responsibility, self-efficacy, or investment in the therapeutic process [38].

It is unsurprising that caregivers who were more actively involved in treatment were those of children who completed treatment. This finding aligns with previous research emphasizing the critical role of caregiver involvement in adolescent substance use treatment, which has been shown to improve treatment adherence, reinforce positive behaviors, and to help identify early signs of relapse [3]. This underscores the importance of minimizing barriers and promoting factors that support caregiver engagement, as caregiver involvement is crucial for promoting successful treatment outcomes.

Caregivers of children who completed treatment often reported increased substance use monitoring, including administering urine drug screens at home. While concerns exists regarding potential privacy breaches, boundary issues between caregivers and their children, and the possibility of conflict within the parent-child relationship [39, 40], structured treatment protocols – such as multisystemic therapy and multicomponent therapy incorporating motivational interviewing, cognitive behavioral therapy, and contingency management – may help to address these challenges [41–43]. These protocols support caregivers in developing skills in interpreting urine drug screens, communicating effectively about substance use, and consistently applying rewards and consequences in line with contingency management principles. By offering clear guidance, they can enhance caregiver confidence and foster as supportive approach to monitoring that strengthens both treatment engagement and family relationships.

### Barriers to caregiver involvement

There were no differences between caregivers of children who completed treatment and those with children who did not complete treatment in their descriptions of structural barriers that hindered treatment engagement. Consistent with the Barriers to Treatment Model [8] and the broader literature on barriers to accessing mental health treatment [44], caregivers reported that the clinic location and limited hours of operation made attending sessions challenging. These findings highlight the need for flexible scheduling and accessible treatment options to support caregiver involvement and overall treatment engagement. Although expansions of SUD services through additional clinic locations or extended hours is possible, both changes would require significant investment from organizational and policy stakeholders to ensure appropriate staffing and long-term sustainability.

Caregivers of children who completed treatment also noted specific child-level factors that made caregiver involvement difficult. Consistent with the Health Belief Model [6, 7], a key barrier to caregiver involvement was caregivers’ struggle to balance supporting autonomy with addressing the child’s low motivation for treatment. Although not identified as a primary theme in the current study, the tension between respecting a child’s autonomy and addressing their low motivation for behavior change or treatment can increase stress and burden for caregivers, who may need to expend additional effort to facilitate child’s participation and engagement in treatment [13, 45]. Including individual caregiver sessions, where the caregiver has their own dedicated therapist to provide emotional and behavioral support, is a promising strategy for reducing stress and burden. In this study, such sessions helped caregivers process their own stress, develop effective strategies for supporting their child, and engage more effectively in their child’s treatment.

### Facilitators to caregiver involvement

Caregivers of children who completed treatment were far more likely to describe logistical factors that supported their involvement in treatment. This pattern is understandable, as caregivers of children who discontinued treatment had fewer opportunities to engage, but it highlights the importance of fostering caregiver involvement early in the treatment process. Aligned with the Barriers to Treatment Model [8], logistical factors such as the option to attend sessions virtually and the ability to schedule medication appointments on the same day as therapy sessions served as key facilitators of caregiver engagement. These logistical supports may help to minimize time, travel, and scheduling challenges for some caregivers, helping to sustain caregiver engagement throughout treatment.

Caregivers of children who completed treatment also highlighted specific aspects of treatment that helped to encourage their involvement in treatment. Consistent with prior qualitative research on the qualities caregivers value most in adolescent substance use treatment [15], strong therapeutic relationships among the provider, caregiver, and child emerged as an important facilitator of treatment engagement. These relationships often fostered trust between the provider and caregiver, which encouraged open communication and enhanced engagement. In line with findings from broader mental health care research [46], psychoeducation also emerged as an important factor for supporting caregiver involvement in adolescent substance use treatment. Psychoeducation may clarify misunderstandings about the consequences and benefits of adolescent substance use, empower caregivers with the knowledge and skills to effectively monitor substance use, and guide them to reinforce coping strategies and healthy behaviors at home [47, 48]. Together these findings suggest that both relational and informational components of treatment are critical for promoting caregiver engagement.

### Limitations

Findings must be understood with respect to several limitations. Aspects of this study likely limit transferability. Interviews were limited to caregivers who agreed to participate in this study and may represent a sampling bias where caregivers who were more engaged in their child’s treatment elected to participate, while those who were less engaged may have opted out of the study. Similarly, these interviews were conducted with caregivers who were able to initiate specialty SUD treatment and may not represent those seeking SUD treatment in general mental health settings. Qualitative thematic analyses also have the potential for research bias, which was mitigated by inter-coder reliability checks and consensus meetings to discuss interpretations with colleagues who are experts in the field of child and adolescent SUD treatment.

The semi-structured interview primarily focused on the motivations for initiating treatment and on caregivers’ involvement in adolescent substance use treatment. Questions regarding caregivers’ own mental health, coping strategies, and self-care were not included in the interview guide and did not emerge organically during the interviews. Similarly, questions did not explicitly ask caregivers about experiences of strain or burden associated with participating in their child’s SUD treatment. As a result, caregiver specific factors that may hinder or facilitate caregiver involvement in adolescent substance use treatment were not fully captured in this study. Future qualitative research should comprehensively explore caregiver wellbeing, coping capacity, and support needs comprehensively to understand how these factors influence engagement in treatment.

## Conclusion

This study offers insights into caregivers’ experiences in adolescent SUD treatment. Although all caregivers were involved in initiating treatment and facilitating attendance, those whose child completed treatment typically assumed more active involvement throughout the course of treatment. Common barriers to involvement included logistical challenges (e.g., clinic location and hours), as well as youth-related factors (e.g., concerns for autonomy, motivation). Adolescent dual diagnosis clinics should incorporate telehealth services and, when feasible, expand physical service locations and hours of operation. Additionally, caregiver education and universal substance use screening may be key components for detecting adolescent substance use early and strengthening connection to care .

## Supplementary Information

Below is the link to the electronic supplementary material.

Supplementary Material 1

## Data Availability

Data are not available to the public to protect the confidentiality of the caregivers and adolescents who completed substance use treatment.

## Abbreviations

CBT: Cognitive Behavioral Therapy
CM: Contingency Management
CRAFT: Community Reinforcement and Family Training
MI: Motivational Interviewing
SBIRT: Screening, Brief Intervention, and Referral to Treatment
SUD: Substance Use Disorder
