# Supplementary material for: A thematic analysis of caregiver engagement in adolescent substance use treatment
Source: Addict Sci Clin Pract. 2026 May 5;21:41. doi: 10.1186/s13722-026-00669-z (PMC13156863; doi:10.1186/s13722-026-00669-z)
Supplement: Supplementary file 1 — Supplementary Material 1 [file 13722_2026_669_MOESM1_ESM.docx]

**ADOLESCENT DUAL DIAGNOSES PROGRAM PARENT ENGAGEMENT
QUALITATIVE INTERVIEW GUIDE**

Thank you for agreeing to participate in our study and for your time in completing this interview with me. Today, I am going to ask you some questions about your beliefs around adolescent substance use treatment, as well as your experience in the Dual Diagnosis Program. As a reminder, this interview will be audio recorded and your responses will be used to both improve the experiences of families in the future who receive treatment in the Dual Diagnoses Program and will be used to inform the development of future family and individual based substance use interventions for adolescents.

**Recognition of Child’s Substance Use**

1. What brought you and [CHILD NAME] to the Dual Diagnosis Program?
   - What, if any, concerns did you have around [CHILD NAME] substance use when you started treatment?
2. When did you first notice [CHILD NAME] was using substances?
3. How did you learn about [CHILD NAME] substance use?
4. What were your initial reactions to [CHILD NAME] using substances?
   - How has your perspective on [CHILD NAME]’s substance use changed over time?
5. To your knowledge, what did [CHILD NAME] substance use look like at its worst?
   - What, if any, worries or fears did you have around [CHILD NAME] substance use when it was at its worst?
6. To your knowledge, what does [CHILD NAME] substance use look like today?
   - How do you know what your [CHILD NAME] substance use looks like today?
   - What, if any, worries or fears do you have around [CHILD NAME] substance use today?
   - What, if any, worries or fears do you have around [CHILD NAME] substance use in the future?

**Caregiver’s Role in Treatment**

1. How can parents or caregivers help children with substance use?
2. Prior to starting in the dual diagnoses program, how did you see your role as a parent in your child’s substance use or substance use disorder treatment?
   - How has this changed since receiving treatment through the Dual Diagnoses Program?
3. Prior to receiving treatment through the Dual Diagnosis Program, did you believe you were able to help [CHILD NAME] with their substance use?
   - How, if at all, has your belief in your ability to help or support [CHILD NAME] with their substance use changed over time?
4. What, if anything, did you try on your own to help [CHILD NAME] with their substance use prior to joining the Dual Diagnosis Program?
5. Tell me about your experience in the Dual Diagnoses program.
   - What was your role in getting [CHILD NAME] to their appointments?
     - What challenges did you face to attending the appointments?
     - What was helpful in attending the appointments?
   - What was your role in adhering to medications?
   - What was your role in supporting your child’s therapy homework (e.g., practicing skills, pro-socials)?
   - What was your experience like attending parent-only sessions?
   - What was your experience like attending family sessions?
6. What, if anything, did you learn during [CHILD NAME]’s substance use treatment?
   - What, if any, specific skills did you learn to help [CHILD NAME]?
   - What, if any, knowledge or skills do you wish you would have learned during [CHILD NAME]’s treatment?

**Barriers and Benefits**

1. How can therapy help treat substance use in children?
   - How can individual therapy for a child living with a substance use disorder be beneficial?
   - How can parent sessions or parent therapy be beneficial for a child living with a substance use disorder?
   - How can family therapy be beneficial for a child living with a substance use disorder?
2. How can medications help with substance use disorders?

- What, if anything, do you know about medications for opioid use disorders?
- What are your beliefs around medications for substance use disorders?
- What are your beliefs about medications for opioid use disorders specifically?

1. What were your initial goals in [CHILD NAME] treatment?
   - - Did your goals change throughout treatment?
     - How did your goals change throughout treatment?
     - Why did your goals change throughout treatment?
2. What did your participation in [CHILD NAME]’s treatment in the Dual Diagnosis program look like?

- Did you participate in parent-only sessions with our parent therapist?
  - Tell me more about that experience.
  - What, if anything, kept you from engaging in parent sessions?
  - What, if anything, encouraged you to engage in parent sessions?
- How many, if any, family sessions did you attend?
  - Tell me more about that experience.
  - What, if anything, kept you from engaging in family sessions?
  - What, if anything, encouraged you to engage in family sessions?
- Did you complete drug screenings at home during [CHILD NAME]’s treatment?
  - [if yes] Tell me more about that experience.
  - [if yes] How often? Were they random? Did you purchase any drug screens yourself in addition to the ones provided by the program?
  - [If No] What, if anything, kept you from completing drug screenings at home?
  - [If No] What, if anything, encouraged you to engage in drug screenings at home?
- Sometimes families connect specific rewards to negative urine screen results. We call this “contingency management” because earning rewards – such as privileges or prizes – is contingent on having a negative screen or meeting some other goal that the family agrees on. Did you utilize this kind of contingency management strategy at home?
  - - Tell me more about that experience.
    - What, if anything, kept you from completing utilizing contingency management at home?
    - What, if anything, encouraged you to utilize contingency management at home?

1. What changes, if any, did you notice after [CHILD NAME] completed treatment?
2. How would you know if [CHILD NAME] should re-engage in treatment?
